# From passive to participatory: exploring pharmacy students’ experiences and perceptions with active learning

**DOI:** 10.1186/s12909-025-08470-3

**Published:** 2025-12-19

**Authors:** Hege Sletvold, Linda Amundstuen, Daniel H. Zeiss, Marcel Köhler

**Affiliations:** Faculty of Nursing and Health Sciences, Nord university, PO Box 474, Namsos, 7801 Norway

**Keywords:** Healthcare education, Pharmacy education, University pedagogy, Competency development, Student-centered learning, Active learning, Peer-learning, Student motivation

## Abstract

**Background:**

Recent legislative reforms in Norwegian education policy have heightened the professional demands on healthcare personnel, including pharmacists. These reforms necessitate adjustments in education, particularly in pedagogical approaches. Student-centered, active learning has emerged as a significant educational strategy to enhance critical thinking, problem-solving, teamwork, and reflective capacities among healthcare students. The aim of this study is to examine the perceptions and experiences of pharmacy students with active learning and what influenced the students’ learning during the activities.

**Methods:**

This study employed a qualitative design using focus groups to explore pharmacy students’ perceptions and experiences with active learning. Conducted at the Faculty of Nursing and Health Sciences, Nord University, Norway, the study involved Bachelor of Pharmacy students. A purposive sampling strategy invited all students in the program to participate. Data was collected through four focus group interviews held between March 2023 and January 2024, using a semi-structured interview guide. Thematic analysis was applied to analyse the data.

**Results:**

Twenty-one students participated, including 4 males and 17 females, that were third year (*n* = 12), second year (*n* = 5), and first-year (*n* = 4) pharmacy students. The analysis revealed three main themes: social constructions, organisation and structure, and transfer value. Social constructions emphasised the role of individual attributes and social dynamics in learning. Organisation and structure highlighted the impact of time management, activity design, and infrastructure. Transfer value focused on the relevance of learning activities to students’ motivation and engagement.

**Conclusions:**

The study demonstrates that active learning in pharmacy education is influenced by a complex interplay of factors affecting both the learning process and outcomes. Key factors include social dynamics, organisational aspects, and the perceived relevance of activities. These findings underscore the need for flexible, student-centered, participatory, and well-structured instructional designs. As pharmacy education continues to adapt to changing professional demands and societal expectations, these insights offer a timely and actionable foundation for fostering reflective, engaged, and practice-ready graduates.

**Trial registration:**

Not applicable.

**Supplementary Information:**

The online version contains supplementary material available at 10.1186/s12909-025-08470-3.

## Background

In recent years, Norwegian education policy has introduced legislative reforms to address the growing professional demands placed on healthcare personnel, as well as the increasing knowledge and skill requirements for pharmaceutical professionals [1–3]. These reforms have significantly influenced existing pharmacy programs. Alongside efforts to adjust the overall framework and content of these programs, there is a pressing need to adapt the methodological and pedagogical approaches used in individual courses. In this context, active learning has emerged as an increasingly significant approach to modern education [4]. By incorporating active learning activities, study programs aim to support critical thinking, problem-solving abilities, foster effective teamwork, and enhance reflective capacities among future pharmaceutical professionals.

Rooted in the principles of constructivism [5], active learning represents a key component of the broader paradigm known as student-centered learning. Constructivist theory emphasises the importance of learning through active engagement and reflective practice, aligning with active learning core objective: to immerse students in hands-on activities that promote both action and critical thinking, including cognitive actions (e.g. planning, reasoning, drawing conclusions) [5, 6]. Active learning can be defined as an instructional strategy and a set of instructional practices designed to foster students’ deliberate engagement with the learning content through processes of thinking, experimentation, application, discussion, and reflection [6]. Unlike modes of education that rely primarily on listening and rote memorisation, active learning encourages students to participate in activities that foster deeper cognitive engagement. These activities are structured to enhance the development of higher-order thinking skills, including analysis, synthesis, and evaluation [4, 6]. Central to active learning is the shift from teacher-centered transmission of information to a model that prioritises students’ active exploration and discovery [7]. In contrast to the traditional direct instruction approach, where the focus is on the direct transmission of knowledge, active learning emphasises the dynamic manipulation and processing of information by learners. While both direct instruction and active learning can achieve similar educational goals, research suggests that active learning environments yield deeper and more equitable learning outcomes. Studies have shown that active learning not only enhances the depth of understanding but also fosters greater inclusivity, ensuring broader engagement among diverse student groups [4, 8, 9]. By promoting experiential learning and cognitive reflection, active learning equips students with the skills necessary for problem-solving, adaptability, and lifelong learning [10].

In a project funded by the Norwegian Directorate for Higher Education and Skills, a needs-oriented spiral curriculum was developed and implemented at Nord University between 2022 and 2024 to promote active learning among students in pharmacy education. The curriculum development approach for medical education served as a theoretical basis [11]. On this basis, numerous active learning activities were developed and implemented in regular lessons, while traditional lectures continue to form an integral part of the courses. All student cohorts were engaged in in-class discussions on active learning, explored its meaning and implications, reflected on it, and collaboratively developed a mutual understanding.

Active learning activities included peer mentoring, a student-led interdisciplinary pharmacy conference, various forms of peer assessment, and structured collaborative techniques such as “think-pair-share,” “team-based learning,” “jigsaw,” and “stop and switch” activities. Several of these activities focused on peer-learning serving as a renowned example of active learning in health professional education that fosters collaboration and knowledge exchange among students [12–15]. The design of these strategies was inspired by established practices in medical and pharmacy education, where active learning has been shown to enhance engagement, critical thinking, and professional identity formation (see e.g., 12, 13). The project aimed to better align the teaching approach in pharmacy studies with the requirements associated with the legal reforms mentioned.

This study will examine the perceptions and experiences of pharmacy students with active learning and what influenced the students’ learning during the activities.

## Methods.

This study employs a qualitative design utilising focus groups to facilitate discussions among study participants on a given topic. The dynamics and interactions within the groups provide insights into the diverse perspectives of the participants and have the potential to generate new ideas and deeper understanding [16]. Consequently, this design is appropriate, offering an opportunity to collect in-depth data on the study aim.

The COnsolidated criteria for REporting Qualitative research (COREQ) checklist was used as a guide for explicit and appropriate reporting [17].

### Research team and reflexivity

The team consists of researchers with extensive experience within social pharmacy, pharmacology, pharmaceutical sciences, pedagogy and health professions education. All have extensive teaching experience from higher education. DZ, HS and LA are pharmacists and educators within the Bachelor of pharmacy program, where the study participants are students. Hence, the participants knew these researchers as teachers, however, the participants did not have a prior relationship with MK. MK is professor for pedagogy of professions at the faculty of Nursing and Health Sciences and holds a PhD in vocational pedagogy. He is involved in faculty development within teaching methodologies, is a state-approved nurse, and is a qualified Senior Teacher for Vocational Schools in the field of Nursing and Health Care.

The researchers represent distinct scientific areas and varying preconceptions on the research topic, enriching the discussions. However, we share an interest in student active learning and education research and development. HS, LA, and MK have experience with qualitative research. HS and LA are female researchers, DZ and MK are male.

### Study setting

This study was done at the Faculty of Nursing and Health Sciences, Nord University, campus Namsos, that hosts students within pharmacy, nursing, paramedics and social education. Bachelor of Pharmacy is provided at campus which admits approximately 30 students per year. The bachelor program is full time, 180 ECTS (European Credit Transfer and Accumulation System) over three years, and includes courses within chemistry, biosciences, pharmaceutical technology, and social pharmacy. A placement period of four months in a pharmacy is mandatory. With a bachelor’s degree in pharmacy, you can, in Norway, apply for authorisation as a “reseptarfarmasøyt”, also called prescriptionist. Prescriptionists are healthcare professionals with specialist expertise relating to the manufacture, quality assurance, use and effects of medicines. They commonly work in community pharmacies.

### Participant selection and recruitment

A purposive sampling strategy was used, where all students at the Bachelor of Pharmacy program at Nord University were invited to participate. They were recruited through written invitations distributed through the university’s web-based learning management system (Canvas), and by face-to-face oral invitation during lectures. Written information about the study, including the aim, explanations about focus groups, data collection, data management, consent, the research team, and the opportunity to withdraw from the study at any time, was provided during the recruitment.

### Data collection

The focus group interviews were planned to take place after specific learning activities, with one focus group per academic year. Third-year students were invited to participate in two separate focus groups, as they had experienced the greatest number of active learning activities. Students were informed about the time and location of the interviews both verbally and via the Canvas learning platform. Participation was voluntary, and students signed up in advance. In total, four focus group interviews were conducted as planned. These were held at Nord University, campus Namsos, in March and December 2023, and January 2024. Participants included pharmacy students in their 1 st, 2nd or 3rd year of study. A semi-structured interview guide was used (Supplementary material 1), developed by HS, LA, and MK. The guide was not pilot tested.

General information about the study and information about what participation entails was given, and written informed consents were given, prior to starting the interviews.

The interviews were moderated by HS, a research assistant was secretary and took notes, and MK was observer. The topic of the interviews were perceptions and experiences with active learning activities that the students had been exposed to, and what is important for student’s learning, according to the interview guide. The interviews were audio recorded and transcribed verbatim by LA and a research assistant, or digital transcripts were produced by Nettskjema, and subsequently checked for accuracy and corrected by LA. The transcripts were not returned to participants.

### Data analysis

The data was analysed through an inductive approach using thematic analysis [18]. The researchers (HS, LA, DZ, and MK) familiarised themselves with the data by reading through all transcripts. Initial codes were generated individually by the researchers using one transcript in detail, then these codes were discussed between the researchers, subsequently searching for themes. Firstly, the themes were manifest. LA reviewed the themes, by checking if the themes worked in relation to codes and coded all transcripts in detail. The development of the themes was achieved in ongoing discussions between all researchers. The final themes created were latent, going beyond the semantic content of the data. The final naming of the themes and the overall story the analysis tells, were defined in collaboration between the researchers. LA drafted the result text for each subtheme, and HS, DZ and MK discussed the text and revised it. The selection of the quote examples was discussed by all researchers and jointly decided. Microsoft 365 Excel was used to support the organisation of codes, subthemes, and themes during the analysis process.

## Results

In total, 21 students participated in the study, four male (19%) and 17 (81%) female. Third year pharmacy students (*n* = 12) participated in two focus groups, and first (*n* = 4) and second year (*n* = 5) pharmacy students participated in one focus group, respectively.

**Fig. 1:**
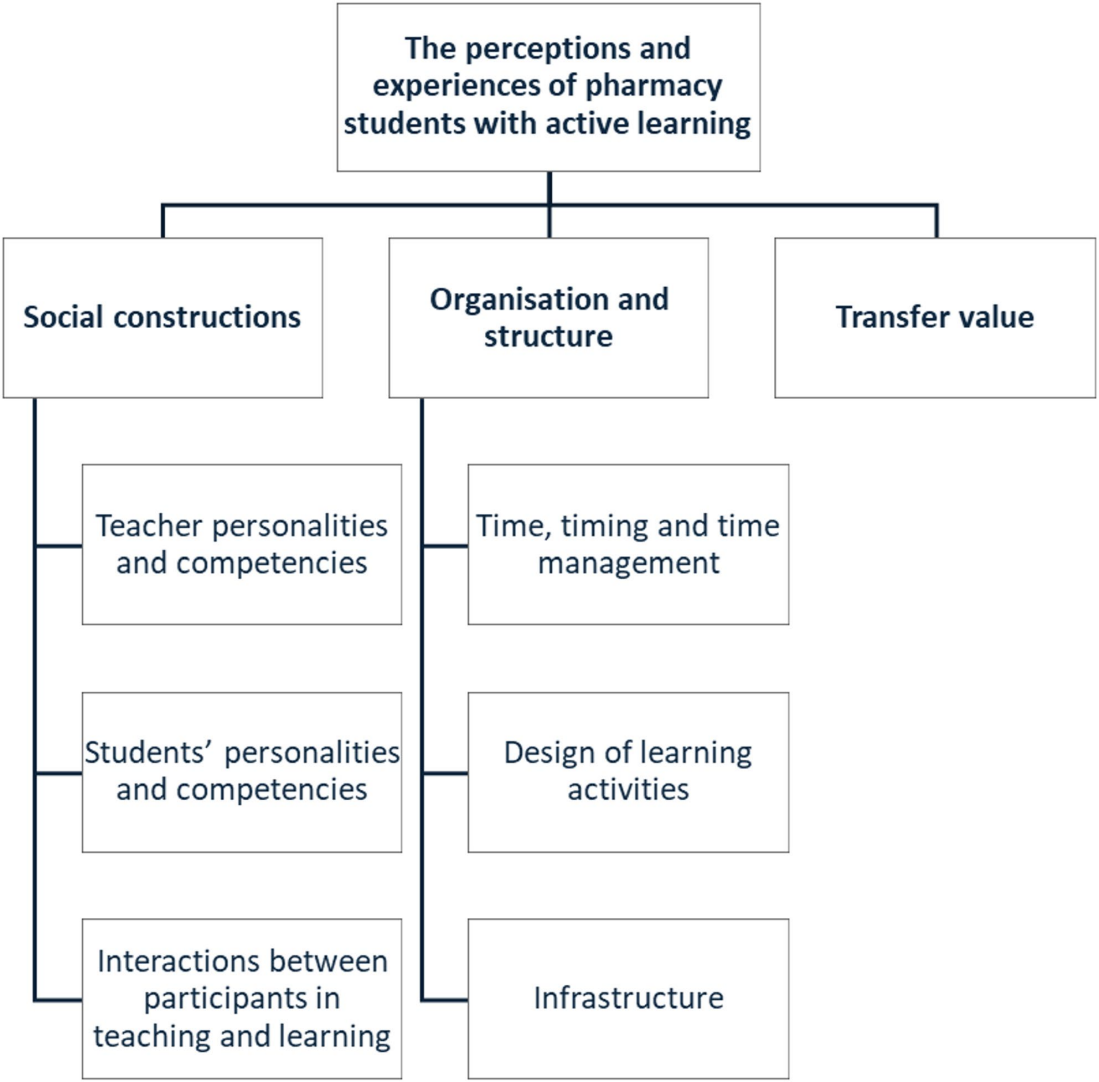
The themes and subthemes describing the students’ experiences with active learning

The perceptions and experiences of pharmacy students with active learning and what influenced the students’ learning during the activities are described through themes and subthemes, where the three main themes were: social constructions, organisation and structure, and transfer value (Fig. 1), see also Supplementary material 2.

### Social constructions

The theme of “Social constructions” explores how the personalities, competencies, and interactions of both students and teachers shape the learning environment and outcomes. The engagement and quality of these interactions influence the emotional experiences and perceived effectiveness of learning activities. This theme highlights the importance of individual attributes and social dynamics in fostering a conducive educational atmosphere.

#### Teachers’ personalities and competencies

 This subtheme delves into how the characteristics and skills of teachers shape students’ perceptions of the learning environment, process, and outcomes. Teachers’ attitudes towards education, their enthusiasm, and their ability to engage and organise learning activities play a crucial role in influencing students’ experiences. The way teachers present information, instruct, and interact with students can significantly enhance the learning atmosphere, making it more engaging and effective.

“The teacher really showed interest in their field. Like yesterday, during the presentation—you were drawn in. The teacher was so engaged and excited to talk about the topic that you just wanted to hear more. It was like, ‘Okay, tell me more!’” (Student 1).

#### Students’ personalities and competencies 

 This subtheme describes how individual characteristics, learning preferences, learning strategies, discipline and motivation influence the learning process and outcomes. Students’ engagement in specific learning activities, as well as their overall approach to learning, impacts their educational experiences. The support from peers and teachers also play a role in fostering a positive and safe learning environment.

*“For me*,* it’s important to always have someone to ask if I’m unsure about something—whether it’s a lecturer or a fellow student. It’s about not being alone*,* having someone around. And it’s also important to be allowed to try and fail—to learn through trial and error. Then*,* with some support*,* you can eventually say*,* ‘Yes*,* I can manage this!’”* (Student 14).

#### Interactions between participants in teaching and learning

 This subtheme describes how the attitudes, knowledge, skills, and engagement levels of both students and teachers shape the social dynamics within educational settings. Effective peer learning activities rely on the active participation and responsibility of each student, while the quality of interactions between students and teachers influences the learning process.

*“We started from the beginning and read through it together. Then she [the fellow student] tested my understanding. If I misunderstood something*,* she didn’t just tell me the answer—instead*,* she gave me some keywords to help me connect the ideas and find the common thread myself. That really helped me when creating the infographic poster.”* (Student 1).

### Organisation and structure

The theme of “Organisation and Structure” encompasses various aspects that influence students’ learning experiences and outcomes. Key elements include the allocation and management of time, the design and arrangement of learning activities, and the structural factors that underpin the educational environment. These components collectively shape the effectiveness of active learning activities, impacting students’ engagement, understanding, and overall academic performance.

#### Time, timing and time management

 This subtheme addresses how the allocation and scheduling of activities impact students’ learning experiences and outcomes. The amount of time dedicated to tasks, the timing of activities in relation to other coursework and exams, and effective time management are important for facilitating students’ learning and reflection. There must be enough time to perform the activities in the way it is organised and allowing for subsequent learning and reflection. Furthermore, time management may include addressing the need for effectiveness of teaching activities. Ensuring that activities are well-organised and efficiently presented can enhance the learning process by preventing time wastage and promote engagement.

*“I often feel that some activities can become time-consuming. I prefer things to be presented in a clear and structured way from the start. While I agree that working through material on your own can also support learning*,* having the content presented directly is much faster—and that saves a lot of time.”* (Student 8).

#### Design of learning activities

The design, organisation, instructions, and follow-up of learning activities affect students learning experiences and outcome. The arrangement of various didactic elements within complex activities, including individual and group work, the complexity of assignments, and the feedback provided by teachers and peers, play a role in shaping the perceived learning outcome of these activities. Effective design ensures that all participants are actively involved and that the process is coherent.

*“This was an assignment that couldn’t really be divided into parts—you had to go through all the steps to reach the final result. So it was important that everyone was involved and contributed. We worked well together on this task. Student 9 and I were in a group with two others.”* (Student 14).

#### Infrastructure

 This subtheme addresses how structural elements within the educational environment impact students’ learning experiences and outcomes. Factors such as the language and complexity of the syllabus, the requirements for attending and assignments, and the alignment of tasks with final exams play a role in shaping students’ perceptions of their learning. Additionally, a lack of coordination among learning activities can create competition between courses, posing a barrier to effective learning.

*“Do you know how long that Danish book have been used in the program? I was just thinking —if it’s going to be used in many years to come*,* why not just have it translated?* (Student 11)

### Transfer value

Students perceived relevance of the learning activities and the topics being covered, affect how they experience the activities, and their motivation for participation. For some students, this motivation grows when activities are seen as relevant to upcoming exams. Additionally, considering activities relevant for future professional practice, affect perceived learning process and outcome.

*“I thought the task we were given today was quite complex—but beautiful. It made you think in different ways and pay attention to all the small details. I wish we had been given a similar task earlier. It was a great opportunity to practise. I felt like I was actually standing in a pharmacy*,* facing a real-life problem. I had to step into the role of a pharmacist*,* read up*,* and be precise.”* (Student 13).

## Discussion

Active learning has been widely recognised in health professions education for its potential to improve student engagement, critical thinking, and collaborative skills [5–7, 19, 20]. However, previous studies have often focused on isolated interventions or general principles, with limited exploration of how students perceive and experience a variety of active learning strategies within a pharmacy curriculum. This study addresses that gap by providing an in-depth qualitative analysis of pharmacy students’ experiences with multiple active learning approaches implemented as part of a broader curricular reform.

The results of this study illustrate that active learning is influenced by a complex combination of various factors, which affect both the learning process and the learning outcomes of the students. In particular, the heterogeneity in the perception of individual learning activities underscores the need to design teaching and learning in a differentiated, flexible, and participatory manner.

A central finding of the study is that social constructions, the organisation and structure of activities, as well as their perceived relevance and transfer value, are essential factors influencing the learning process and learning outcomes. These insights confirm and extend existing findings from higher education research, which indicate that not only the activity itself but also its didactic embedding, the quality of interaction, and the applicable relevance are decisive [19, 20]. Future research should explore how learning activities can be planned to accommodate differing student perceptions, in line with constructivist and active learning principles [5–7]. Furthermore, small changes in the organisation of learning activities, e.g., in the temporal sequence, task distribution, or supervision, can make a considerable difference in perception and learning experience [7]. This highlights that instructional design requires high attention and sensitivity not only in terms of content but also organisationally.

A particularly noteworthy result of this study is that student’s motivation and willingness to learn are strongly aligned with the practical relevance of the tasks, either in relation to upcoming examinations or future professional requirements. Self-Determination Theory (SDT) offers a robust explanatory framework for these findings. According to SDT, the quality of students’ motivation is shaped by the extent to which learning environments support their basic psychological needs for autonomy, competence, and relatedness [21]. In the context of pharmacy education, experiential and practice-based learning activities often address these needs directly: students exercise professional judgment (autonomy), receive feedback on authentic tasks (competence), and engage with preceptors, peers, and patients (relatedness). Prior research has argued that supporting these psychological needs within a professional pharmacy curriculum can foster the development of professional identity and better prepare students for diverse healthcare settings [22]. This underscores the importance of designing learning activities that are not only content-rich but also transparent in purpose and pedagogical intent. It is advisable for instructors to clarify their choice of methods and objectives to convey meaning and benefit. Furthermore, these findings underline the need for pharmacy educators to adopt instructional design practices that are both pedagogically sound and responsive to students’ evolving professional identities. To enhance the perceived relevance and effectiveness of active learning, educators may benefit from clearly linking chosen methods – such as small-group case work, stop and switch, and the jigsaw method – to students’ learning goals and professional development. Clarifying instructional design approaches, offering multiple opportunities for reflection, and explicitly communicating the connections between learning activities, assessments, and pharmacy practice can foster deeper engagement and motivation. To balance exam relevance with professional practice orientation requires thoughtful integration of assessment strategies. Educators might consider aligning assessments with professional competencies, scaffold complexity across the curriculum, and combining traditional tests with practice-based tasks. Frequent formative feedback could be supportive tools for educators to aid students’ learning and reinforce the relevance of active learning to both academic success and future professional roles as pharmacists.

The assumption of responsibility in cooperative settings also proves to be substantial. The data suggest that learning in peer-learning situations depends not only on individual commitment but also crucially on mutual involvement and responsibility. This is also indicated by other studies [4, 8]. Moreover, a peer learning and assessment model tested in an undergraduate pharmacy course found that it increased the student’s enthusiasm and ability to learn, and promoted collaborative learning [15]. These findings point to a dual challenge: students need motivation, self-discipline, and social skills, while educators must ensure that the framework conditions for successful group processes are established.

This study underscores the increasing importance of active learning as a pedagogical strategy in response to the evolving requirements of pharmaceutical education in Norway. In light of recent legislative reforms that have redefined requirements for professional competencies [1, 2], pharmacy curricula are compelled not only to revise their content but also to fundamentally reconceptualise their instructional designs. The incorporation of active learning, based on constructivist theoretical frameworks, signifies a substantive transition toward a more individual, engaging, and reflective educational paradigm. This includes fostering a safe, inclusive environment where learning through failure is accepted and supported, and where students are encouraged to take ownership of their learning process. By offering a detailed account of students’ experiences and perceptions, this study provides actionable insights for pharmacy educators and contributes to the broader discourse on how active learning can be effectively integrated into professional education.

### Study limitations

The purposive sampling strategy, involving voluntary participation from 21 students, may have introduced selection bias. It is plausible that students with a particular interest in active learning, pedagogy, or educational research were more inclined to participate in the focus groups. Furthermore, given that members of the research team also held faculty roles, the potential for social desirability bias must be considered. These factors may influence the nature of the responses and limit the generalisability of the findings. While the insights provide valuable perspectives within the studied context, the transferability of results to other institutions or larger cohorts should be interpreted with caution.

## Conclusion and recommendations

This study emphasises the importance of further investigating how social construction, organisational structure, and transfer value collectively influence the learning process and motivation for pharmacy students and other health care education participants. The findings highlight that a supportive social environment and a well-structured organisational setting are crucial for fostering self-directed learning and motivation. This is evidenced by the positive impact of peer influence, role models, and a constructive learning atmosphere on student engagement, learning process, and learning outcomes. Additionally, the value that students place on the transferability of their learning to real-world practice is a significant driver of motivation and professional identity development. For health care lecturers, these insights emphasise the need to design educational experiences that not only impart knowledge but also cultivate the skills and attitudes required for effective collaboration and lifelong learning within dynamic social and professional contexts. For pharmacy educators, these insights suggest several actionable strategies:

- Design learning activities with clear relevance to both exams and pharmacy professional practice, using methods such as case-based learning, simulations, and peer collaboration.
- Clarify instructional design choices and communicate the purpose and expected outcomes of each activity to students.
- Integrate diverse assessment formats, including formative feedback, to support learning and motivation.
- Foster inclusive and relational learning environments, where students feel safe to engage, reflect, and learn from failure.
- Support differentiated learning by accommodating varied student perceptions and needs through flexible and participatory teaching approaches.

By implementing these strategies, educators can better support pharmacy students in developing the competencies, motivation, and collaborative skills needed to thrive in and contribute meaningfully to the dynamic and evolving landscape of healthcare.

## Supplementary Information

Supplementary material 1.

Supplementary material 2.

## Data Availability

The datasets analysed during the current study are available from the corresponding author on reasonable request.
