# Supplementary material for: From passive to participatory: exploring pharmacy students’ experiences and perceptions with active learning
Source: BMC Med Educ. 2025 Dec 19;25:1748. doi: 10.1186/s12909-025-08470-3 (PMC12750662; doi:10.1186/s12909-025-08470-3)
Supplement: Supplementary file 1 — Supplementary material 1. [file 12909_2025_8470_MOESM1_ESM.docx]

**Additional file II**

### Overview of analysis results: Themes and subthemes with example quotes.

| Theme | Subtheme | Quote |
| --- | --- | --- |
| Social constructions | Teachers’ personalities and competencies | “You could basically ask about anything at any time." (Student 18) |
|  |  | "I think the most important thing is that the person who created the activity has put in effort. Because when I look at it now, I can see that they’ve really put work into making this assignment." (Student 5) |
|  | Students’ personalities and competencies | “When I’m learning something, I can’t just be thrown into the material. I often want to understand… It’s like building a house, right? You want a strong wall before you put on the roof.” (Student 11) |
|  |  | “For me to learn, it’s really helpful to have tasks and to practise continuously—just keep doing exercises. That way, you improve by failing and then trying again.» (Student 18) |
|  | Interactions between participants in teaching and learning | "My point is that we’re here to learn—to work with subject material and assignments to understand things. Not… I mean, to work with people who want the same thing, but not with those who just… just complete assignments to submit them, without learning anything." (Student 9) |
|  |  | “For me personally—I know I get along well with the people in my group, and that we know each other’s strengths and weaknesses. I work a bit slowly, for example, and then, if I ask for help, it’s like, “Oh yeah, sure, we can go back and help you with the task you’re struggling with,” because the others might work a bit faster than I do.” (Student 18) |
| Organisation and structure | Time, timing and time management | “Sometimes it’s like—if there are several group assignments at the same time, or if no time is set aside for them—it can feel like you don’t really have time.” (Student 9) |
|  |  | **“**I’m a bit time-sensitive. In a busy everyday life. So, when I’m here [at the university], I expect things to run smoothly.” (student 7) |
|  | Design of learning activities | And there was another thing I thought worked really well—before we had that supervision session, we acted out all sorts of situations. I found that really helpful, because we had already rehearsed what to do if this happens, or if that happens. I thought it was very helpful» (Student 7) |
|  |  | Student 2: We should do more peer assessments, so that we actually learn what to evaluate over time. Student 3: Yes. Student 1: That way, you get to know the expectations and what’s required. Student 2**:** Mhmm. Student 3: I think we were given an answer key, but how much of the students’ written text needs to match the key to be approved? (Student 1, 2, and 3, in discussion,) |
|  | Infrastructure | “I really like the curriculum books, even though they can be quite heavy sometimes. Several of them are very heavy. That might be one of the reasons they’re not always used—because they’re simply too demanding for us.” (Student 2) |
|  |  | “Organic chemistry was mentioned earlier, and in that subject, we had many mandatory assignments. It’s the subject where I feel I’ve made the most progress, because there were tasks we had to complete. Even though it was a bit frustrating to do all those assignments, it paid off in the end.” (Student 16) |
| Transfer value | | “I want to do as well as possible on the exam so I can become the best pharmacist I can be. Yes, I want to do well so that I actually learn what I’m supposed to learn—and remember it afterwards.” (Student 3) |
|  |  | “… so just learning how to supervise others—that’s something we need. But it’s not quite the same, because we’re not… we might talk about the body, but mostly we talk about medicines [in pharmacies]. Still, we need to learn the art of supervision and giving advice.” (Student 1) |
