# Supplementary material for: From passive to participatory: exploring pharmacy students’ experiences and perceptions with active learning
Source: BMC Med Educ. 2025 Dec 19;25:1748. doi: 10.1186/s12909-025-08470-3 (PMC12750662; doi:10.1186/s12909-025-08470-3)
Supplement: Supplementary file 2 — Supplementary material 2. [file 12909_2025_8470_MOESM2_ESM.docx]

**Additional file I**

## Interview Guide for Focus Group with Students

**It is assumed that written informed consent has been obtained in advance.**

1. **Introduction (10-15 minutes)**
   - Welcome the participants, briefly introduce the project and the interview participants.
   - Explain what will happen during the interview.
   - Inform the participants that the conversation will be recorded and that all personal data will be anonymised.
   - Start the audio recording!
2. **Main Part (30-40 minutes)**
   - **General Start (ask all participants in turn)**
     - What does a good teacher mean to you?
     - What is important to you when attending learning activities?
     - What collaborative learning activities do you remember attending? Can you tell me a bit about them?
   - **Experience with Learning Activities (discussion)**
     - What have you experienced when participating in the learning activities (collaborative learning/peer assessment)?
       - Can you describe what you have done?
       - How have you experienced these activities so far in your studies?
       - What has been challenging about these activities?
   - **Experience with Other Students (discussion)**
     - You have conducted these learning activities together with other students. What experiences have you had regarding:
       - Communication – how did this work? What did you learn?
       - Collaboration – how did this work? What did you learn?
       - Supervision – how did this work? What did you learn?
   - **Impact of Activities (discussion)**
     - Which of the activities would you say have had the greatest impact on you?
       - Are there any activities that have been more or less useful or meaningful than others?
   - **Future Impact (discussion)**
     - How will this experience affect you as a student in the future?
   - **Relevance to Pharmacy Work (discussion)**
     - By participating in the learning activities (collaborative learning/peer assessment), what have you learned that could be useful in your work as a pharmacist?
3. **Summary (5-10 minutes)**
   - The secretary and moderator summarise the notes and provide a brief summary.
4. **Closing Questions (5-10 minutes)**
   - Is there anything you would like to add, or correct?
   - Any thoughts during the interview that you feel have not been addressed?
5. **Conclusion**
   - Turn off the audio recorder. Thank the participants for their participation.
